# Evaluation of Bi-Layer Silk Fibroin Grafts for Penile Tunica Albuginea Repair in a Rabbit Corporoplasty Model

**DOI:** 10.3389/fbioe.2021.791119

**Published:** 2021-12-07

**Authors:** Gokhan Gundogdu, Zhamshid Okhunov, Stephanie Starek, Faith Veneri, Hazem Orabi, Sarah A. Holzman, Maryrose P. Sullivan, Antoine E. Khoury, Joshua R. Mauney

**Affiliations:** ^1^ Department of Urology, University of California, Irvine, Irvine, CA, United States; ^2^ Department of Urology, Children’s Hospital of Orange County (CHOC), Orange, CA, United States; ^3^ Department of Surgery and Harvard Medical School, Boston, MA, United States; ^4^ Division of Urology, Veterans Affairs Boston Healthcare System, West Roxbury, MA, United States; ^5^ Department of Surgery, Brigham and Women’s Hospital, Boston, MA, United States; ^6^ Department of Biomedical Engineering, University of California, Irvine, Irvine, CA, United States

**Keywords:** biomaterials, silk fibroin, corporoplasty, tissue engineering, regeneration

## Abstract

The use of autologous tissue grafts for tunica albuginea repair in Peyronie’s disease and congenital chordee is often restricted by limited tissue availability and donor site morbidity, therefore new biomaterial options are needed. In this study, bi-layer silk fibroin (BLSF) scaffolds were investigated to support functional tissue regeneration of tunica albuginea in a rabbit corporoplasty model. Eighteen adult male, New Zealand white rabbits were randomized to nonsurgical controls (NSC, *N* = 3), or subjected to corporoplasty with BLSF grafts (*N* = 5); decellularized small intestinal submucosa (SIS) matrices (*N* = 5); or autologous tunica vaginalis (TV) flaps (*N* = 5). End-point evaluations were cavernosography, cavernosometry, histological, immunohistochemical, and histomorphometric assessments. Maximum intracorporal pressures (ICP) following papaverine-induced erection were similar between all groups. Eighty percent of rabbits repaired with BLSF scaffolds or TV flaps achieved full rigid erections, compared to 40% of SIS reconstructed animals. Five-minute peak erections were maintained in 60% of BLSF rabbits, compared to 20% of SIS and TV flap reconstructed rabbits. Graft perforation occurred in 60% of TV group at maximum ICP compared to 20% of BLSF cohort. Neotissues supported by SIS and BLSF scaffolds were composed of collagen type I and elastin fibers similar to NSC. SIS and TV flaps showed significantly elevated levels of corporal fibrosis relative to NSC with a corresponding decrease in corporal smooth muscle cells expressing contractile proteins. BLSF biomaterials represent emerging platforms for corporoplasty and produce superior functional and histological outcomes in comparison to TV flaps and SIS matrices for tunica albuginea repair.

## 1 Introduction

Tunica albuginea defects resulting from penile trauma, cancer, Peyronie’s disease, or penile chordee repair necessitate surgical correction to restore tissue integrity, maintain erectile function, and preserve penile length and girth. Free and pedicled autologous flaps derived from the saphenous vein, tunica vaginalis (TV), temporalis fascia, and dermis are used clinically to repair the tunica albuginea and underlying corpora cavernosa as a result of corporoplasty (Khope and Sirsat, 2004; Macedo et al., 2013). The choice of graft material depends on the surgeon expertise, extent of penile deformity, and baseline erectile function (Carson and Levine, 2014). Nonetheless, all these approaches have significant drawbacks including donor site morbidity, limited tissue availability, and potential for vascular injury (Perovic et al., 2009; Lakhiani et al., 2016). Decellularized tissue grafts derived from small intestinal submucosa (SIS), dermis, cadaveric pericardium, and tunica albuginea as well as synthetic meshes such as Dacron and GoreTex have also been investigated as alternative scaffolds for corporoplasty in both animal models and human studies (Chun et al., 2001; Egydio et al., 2002; Knoll, 2002; Wefer et al., 2002; Leungwattanakij et al., 2003; Weiser et al., 2003). Unfortunately, these matrices have been reported to elicit chronic inflammatory reactions, fibrotic tissue remodeling, infection, recurrence of penile curvature, penile length loss, and diminished sexual satisfaction following penile reconstruction (Brannigan et al., 1998; Soergel et al., 2003; Chung et al., 2011). These studies highlight the need for the development of new scaffold designs for tunica albuginea repair capable of promoting intrinsic regenerative responses and preservation of erectile function.

Acellular, bi-layer silk fibroin (BLSF) biomaterials represent promising platforms for corporoplasty given their low immunogenicity as well as tunable structural, mechanical, and degradative properties (Sack et al., 2016). The bi-layer architecture of these protein-based scaffolds has the potential to preserve penile hemodynamics during erectile stimulation via a fluid-tight film layer which prevents blood extravasation from the corpus cavernosa, whereas an annealed porous foam compartment serves as a conduit for host tissue ingrowth at the implant site (Algarrahi et al., 2018a; Algarrahi et al., 2018b; Affas et al., 2019). BLSF grafts have been previously explored for augmentation cystoplasty and urethroplasty in preclinical animal models and were found to support the formation of innervated, vascularized neotissues with functional properties similar to native counterparts (Algarrahi et al., 2018a; Affas et al., 2019; Chung et al., 2014a; Chung et al., 2014b). In addition, parallel comparisons with SIS scaffolds have shown that BLSF matrices display superior biocompatibility in settings of surgical reconstruction with significantly reduced chronic inflammatory reactions in implant regions (Chung et al., 2014b; Algarrahi et al., 2015). In the present study, we hypothesized that BLSF scaffolds will promote superior functional tissue regeneration of tunica albuginea defects in comparison to TV flaps and SIS matrices in a rabbit corporoplasty model.

## 2 Materials and Methods

### 2.1 Biomaterials

BLSF biomaterials were fabricated from aqueous silk fibroin solutions derived from *Bombyx mori* silkworm cocoons and steam sterilized prior to surgical procedures using published protocols (Seth et al., 2013).

To create the BLSF matrix, a silk fibroin solution (8% weight/volume) was dehydrated in a casting vessel for 48 h under laminar air flow to construct a silk fibroin film. An additional silk fibroin solution (6% weight/volume) intercalated with sieved granular NaCl (500–600 μM, average crystal diameter) was layered on the surface of the silk fibroin film to produce a fused porous foam compartment following 48 h of casting at 37°C in a humidified incubator. Residual NaCl was removed from the resulting BLSF graft via washing with distilled water for a period of 72 h. Mechanical and structural properties of BLSF grafts have been reported in previous studies (Algarrahi et al., 2018b). Briefly, BLSF grafts contained a foam compartment with interconnected pores (∼400 μm diameter) annealed to a non-porous, uniform film layer (∼200 μm thick). Uniaxial tensile properties of the BLSF graft were previously reported as elastic modulus: 3.6 ± 1.3 MPa; ultimate tensile strength: 0.3 ± 0.1 MPa; and elongation to failure: 24.7 ± 8.9% (Algarrahi et al., 2018b). Commercially available, acellular SIS matrices (4-ply, Cook, Bloomington, IN) and autologous TV grafts were evaluated in parallel as points of comparison.

### 2.2 Surgical Procedures

All surgical manipulations, animal husbandry methods, as well as functional and imaging assessments adhered to the National Institutes of Health’s Guidelines for the Care and Use of Laboratory Animals and were reviewed and approved by the University of California, Irvine Animal Care and Use Committee in accordance with protocol AUP-20-077. Eighteen adult male, New Zealand white rabbits (3.5–4 kg, Western Oregon Rabbit Co. Philomath, OR, USA) were randomized into 4 experimental cohorts including nonsurgical controls (NSC, *N* = 3) or those implanted with BLSF grafts (*N* = 5); SIS matrices (*N* = 5); or autologous TV flaps (*N* = 5) following the procedures below. This study was also conducted in compliance with ARRIVE guidelines (https://arriveguidelines.org).

**FIGURE 1:**
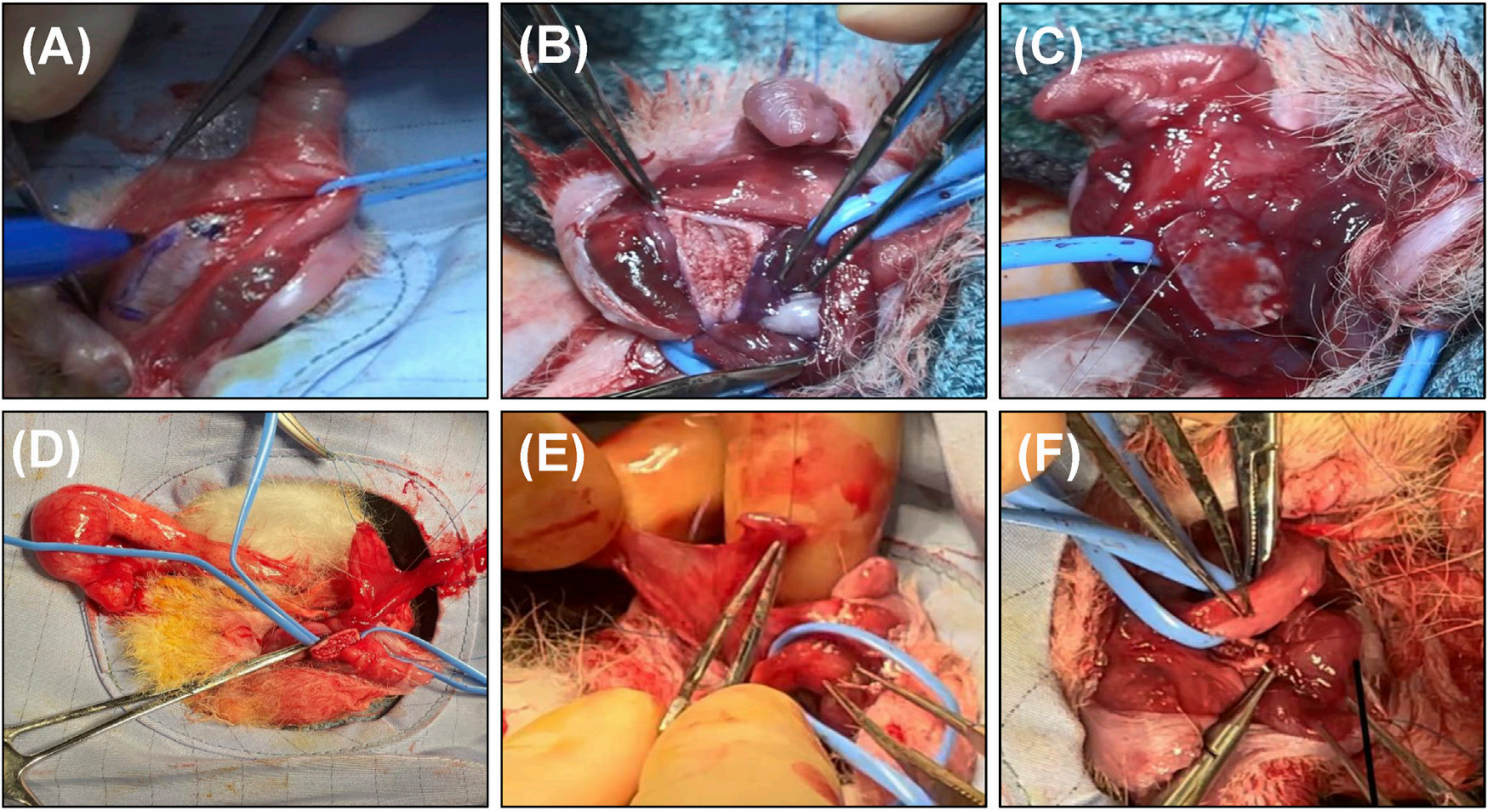
Rabbit corporoplasty model. Overview of surgical reconstruction stages with BLSF grafts **(A–C)** and TV flaps **(D–F)**. **(A)** The penile skin was degloved and the urethra dissected and lateralized from the cavernous bodies to expose the target implantation area. **(B)** Creation of tunica albuginea defect (10 × 7 mm). **(C)** BLSF graft anastomosed into defect. **(D)** Dissection of the TV flap from the anterior surface of the right testes with preservation of the vascular supply followed by transposition through the scrotum and penile skin. **(E)** The distal end of the TV flap was sutured horizontally on its visceral surface to create a patch for tunica albuginea repair. **(F)** Surgical integration of the TV flap into the defect area. BLSF, bi-layer silk fibroin scaffold; TV, tunica vaginalis.

Biomaterial groups and TV flaps were investigated in a previously described rabbit corporoplasty model (Hafez et al., 2001) for a 3 months implantation period (Figure 1). Under isoflurane inhalation and following subcutaneous injection of 35 mg/kg Ketamine and 5 mg/kg Xylazine, animals were placed in the supine position, excess hair was clipped around the surgical site, and the genitalia were scrubbed with a povidine-iodine solution and covered with a sterile drape. A 5-0 polyprolene stay suture was placed at the distal dorsal penile skin adjacent to glans to facilitate penile manipulation. The skin between the penis and anus was divided and the ventral penile skin was incised. Next, two preputial glands over the Buck’s fascia were dissected and the fascia was incised longitudinally to expose the urethra and underlying penile body. At this stage a silicone catheter was introduced into the urethra to assist in dissecting the urethra from the underlying corporal body. The middle segment of the urethra was then completely mobilized from the corporal body and a tourniquet was applied at the base of penile corpora to minimize bleeding.

For rabbits receiving TV grafts, a transverse scrotal incision was made and the TV layer from the right testis was freed from scrotal attachments following penile degloving and urethral mobilization. The anterior surface of the isolated TV was incised longitudinally from both edges while preserving the vascular pedicle. A subcutaneous tunnel was then created between the right scrotum and penile incision and the flap was passed through. The TV flap was sutured horizontally on its visceral surface with 6-0 polydioxanone sutures to create a patch for tunica albuginea repair. In all groups, a 10 × 7 mm (length x width) defect was then created in the tunica albuginea via tissue resection while avoiding injury to the underlying corpora cavernosa. Autologous TV flaps or acellular implants of equal size were anastomosed into the defect site using 6-0 polydioxanone sutures in a continuous locking fashion. Proximal/distal and lateral graft edges were marked with nonabsorbable 6-0 propylene sutures for identification of the original implant site. Skin incisions were then subsequently closed using 6-0 polyglactin sutures.

For pain management, all animals received a single subcutaneous injection of 0.12 mg/kg Buprenorphine SR (ZooPharm, Laramie, WY, United States) immediately after the surgery and daily subcutaneous injection of 1 mg/kg Banamine (Merck Animal Health, Kenilworth, NJ, United States) for 3 days. Animals were also given subcutaneous injection of 5 mg/kg Enrofloaxacin (Baytril®100; Bayer Healthcare LLC, KA, United States) prior to surgery and for 3 days postoperatively to prevent infection. All rabbits wore an Elizabethan collar for 1 week following penile reconstruction to mitigate self-mutilation of the surgical site. Animals were examined daily to assess potential graft complications and sacrificed at 3 months for endpoint analyses described below.

### 2.3 Cavernosography and Cavernosometry

Cavernosography and cavernosometry analyses were carried out in NSC and reconstructed groups following 3 months of implantation using published protocols (Chen et al., 2010). Animals were anesthetized with isoflurane inhalation as well as subcutaneous administration of 35 mg/kg Ketamine and 5 mg/kg Xyzaline and placed in the supine position with a sterile surgical field. The penis was degloved and two 22-gauge IV cannulas were inserted into the proximal right and left cavernous bodies below the original reconstructed area. For cavernosography, contrast medium (Omnipaque 300; GE Healthcare Inc., Marlborough, MA, USA) diluted with 1:1 saline was infused into the cavernous bodies. Anterior/posterior and lateral images were acquired with C-arm fluoroscopy (BV Pulsera; Philips, Eindhoven, Netherlands) to display penile cavernosal anatomy. For cavernosometry, cannulas were connected to a urodynamics system (Goby CT; Laborie, ON, United States) to facilitate evaluations of intracorporal pressure (ICP). Baseline ICP measurements were performed and then heparinized saline (10 U/ml) was delivered into the corporal bodies at a rate of 1–2 ml/min after 1–4 injections of the vasodilator, papaverine (15 mg) to induce penile erection. Maximum ICP values were recorded and maintained for a period of 5 min with saline infusion. Photomicrographs of penile erections were acquired at maximum ICP levels. Rabbits were then sacrificed with an intravenous injection of 0.2 ml/kg pentobarbital sodium and phenytoin sodium euthanasia solution (Euthasol; Virbac AH, Westlake, TX, United States) for subsequent outcome analyses.

### 2.4 Histological, Immunohistochemical, and Histomorphometric Analyses

Penile tissues harvested from NSC as well as implant groups at 3 months post-op were excised for routine histological processing following rabbit sacrifice. Tissue specimens were fixed in 10% neutral-buffered formalin, dehydrated in graded alcohols, and embedded in paraffin using standard protocols. Five micron sections were stained with Masson’s trichrome (MTS) and total collagen content of the corpora cavernosum was determined with an ImageJ color segmentation program *via* quantitation of blue-stained color elements as previously described (Affas et al., 2019). Parallel sections in all groups were also evaluated for the presence of elastin fibers in control and regenerated tunica albuginea using a commercially available, Verhoeff Van Gieson (VVG) staining kit (ab150667, Abcam, Cambridge, MA). Immunohistochemical (IHC) assessments were carried out on tissue sections following antigen retrieval (10 mM sodium citrate buffer, pH 6.0) and incubation in phosphate-buffered saline with 0.3% Triton X-100, 5% fetal bovine serum, and 1% bovine serum albumin for 1 h at room temperature. Sections were then independently probed with primary antibodies including anti-collagen type I (NB600-450, 1:50 dilution, Novus Biologicals, Littleton, CO) and anti-α-smooth muscle actin (SMA) (1:200 dilution; Sigma-Aldrich, St. Louis, MO) for 12 h at 4°C. For collagen type I detection, samples were stained with species-matched horseradish peroxidase (HRP)-conjugated secondary antibodies followed by hematoxylin counterstain. For SMA, specimens were incubated with species-matched Alexa Fluor 594-conjugated secondary antibodies (Thermo Fisher Scientific, Waltham, MA) with nuclei counterstained with 4′, 6-diamidino-2-phenyllindole (DAPI). Sample visualization was carried out with a Zeiss Axio Imager M2 model (Carl Zeiss MicroImaging, Thornwood, NY) and representative fields were acquired with Zen software (version 3.1). Negative controls consisting of parallel specimens stained with secondary antibodies alone were performed similarly and produced no detectable background signal. For histomorphometric analyses, quantitation of total collagen content (MTS) and SMA expression was calculated across 2 independent global sections per group replicate and displayed as a proportion of stained area per total field area examined and normalized to NSC values.

### 2.5 Statistics

Statistical analyses of quantitative data were carried out using the Kruskal–Wallis test with the post hoc Dunn’s test for pairwise comparisons using an online web statistical calculator, www.astatsa.com considering a value of *p* < 0.05 as significant. Quantitative data were represented as mean ± standard deviation (SD).

## 3 Results

All rabbits in each group survived corporoplasty procedures and were maintained for 3 months following implantation until scheduled euthanasia. There were no significant differences in mean operative times between experimental cohorts (BLSF scaffolds, 67 ± 13 min; SIS implants, 78 ± 22 min; TV grafts, 83 ± 13 min). Intra-operative complications were limited to one rabbit in the BLSF group which suffered accidental urethral perforation and was repaired with 7-0 polydioxanone sutures without incident. Transient bladder catheterization was performed due to delayed urination in one rabbit from both the TV and BLSF groups at post-operative day 3 and 5, respectively. Rabbits repaired with TV flaps also displayed mild edema and swelling in the reconstructed area and tissue harvest site for the initial 7 days following surgery. All rabbits were capable of voluntary voiding throughout the study period.

**FIGURE 2:**
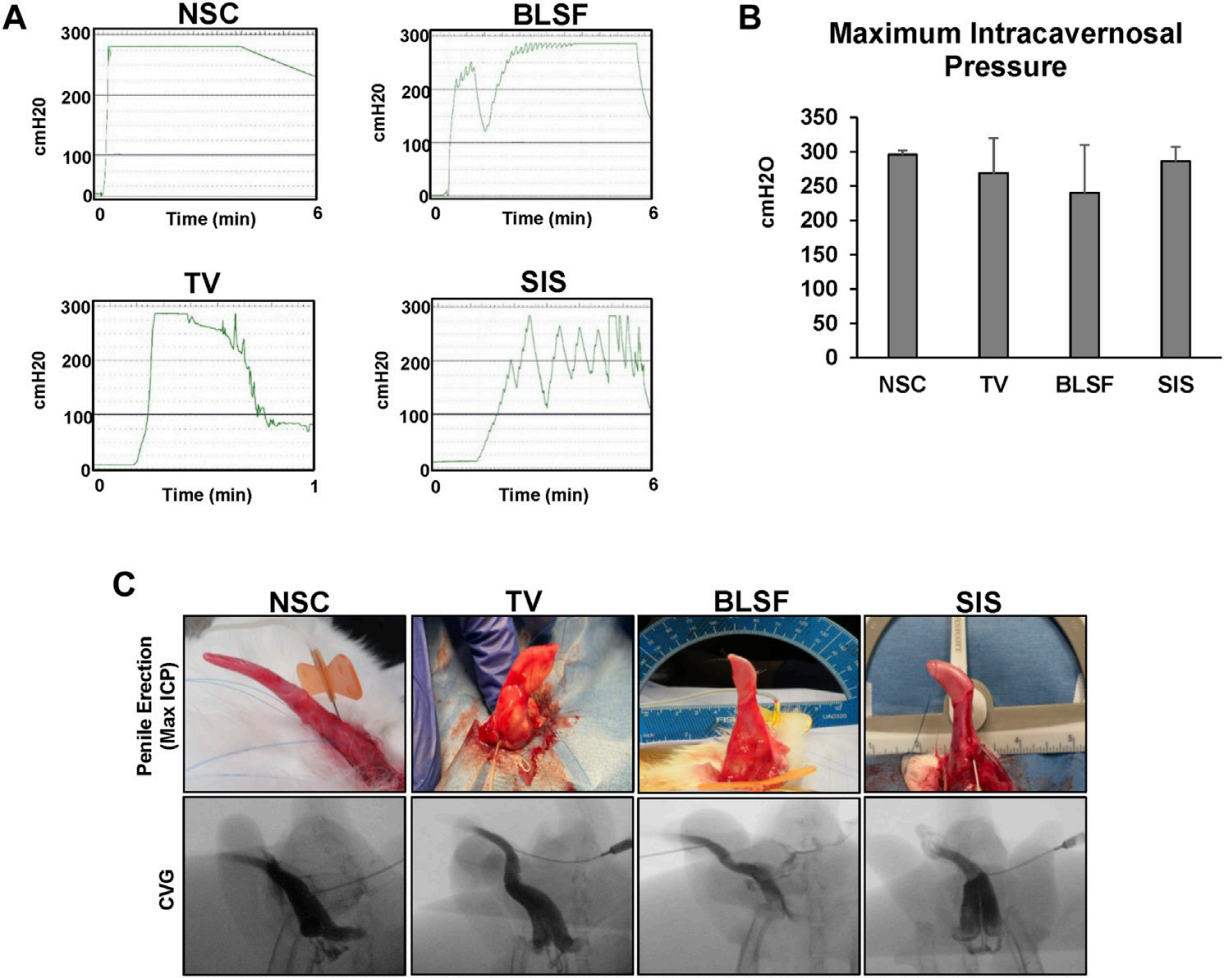
Cavernosometric and cavernosographic evaluations of control and reconstructed penile tissues. **(A)** Representative intracorporal pressure (ICP) tracings from NSC and animals repaired with BLSF, TV, or SIS implants and assessed at 3 months post-op. **(B)** Maximum ICP levels achieved in experimental groups described in **(A)**. Values are presented as means ± SD. *N* = 3–5 replicates per data point. Results from all groups were analyzed with Kruskal–Wallis test yielding *p* > 0.05. **(C)** Representative photomicrographs of penile erections and cavernosographic findings at maximum ICP levels following contrast instillation for groups described in **(A)**. NSC, nonsurgical controls; BLSF, bi-layer silk fibroin scaffold; TV, tunica vaginalis; SIS, small intestinal submucosa.

Penile erectile function was evaluated by cavernosography and cavernosometry in NSC and reconstructed groups at 3 months post-op (Figure 2). Following injection of contrast agent, the corpora cavernosa across all repaired tissues in each group filled with fluid in a homogenous manner in the absence of leakage similar to NSC with the exception of focal radiotranslucent defects centered in the reconstructive areas. Cavernosometric assessments were performed to determine the vascular pressure in the corpus cavernosum following papaverine-induced erection. No significant differences in maximum ICP values were observed between NSC (296 ± 6 cmH_2_O) and experimental groups (BLSF: 240 ± 70 cmH_2_O; SIS: 286 ± 21 cmH_2_O; TV: 269 ± 51 cmH_2_O). Full erections were achieved and sustained for 5 min in 100% of NSC. Rabbits repaired with BLSF grafts and TV flaps demonstrated that 80% of their respective cohorts could achieve full rigid erections at maximum ICP values, in contrast to only 40% of animals repaired with SIS matrices. Moreover, 20% of rabbits implanted with SIS grafts or TV flaps were capable of maintaining a peak erection for the 5 min evaluation period, in contrast to 60% of BLSF replicates. In addition, perforation of the implant area occurred during peak erection in 60% of the rabbits reconstructed with TV flaps, while only 20% of those reconstructed with BLSF matrices displayed deficiencies in neotissue structural integrity. These results provide evidence that corporoplasty with BLSF grafts supports superior erectile function in comparison to surgical approaches with TV flaps and SIS implants which led to higher incidences of perforation and partial, non-sustained erections, respectively.

**FIGURE 3:**
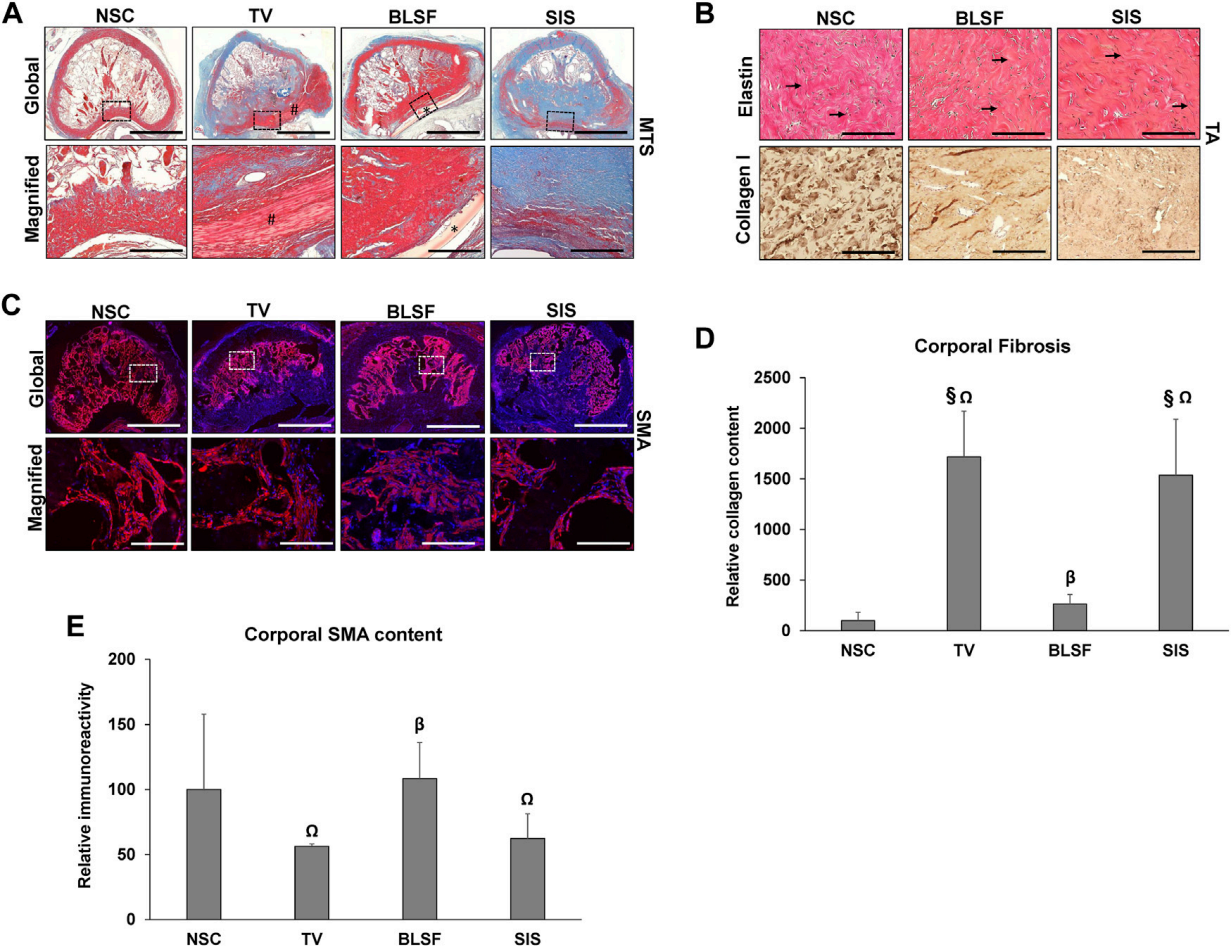
Histological, immunohistochemical and histomorphometric assessments of control and reconstructed penile tissues. **(A)** Representative cross-sectional views of Masson’s trichrome (MTS)-stained, penile tissues repaired with implant groups at 3 months post-op or treated as NSC. Gross cross-sections of penile tissues are presented in the top row with magnified views (boxed) of control or graft sites displayed in the second row. (^#^) denotes autologous TV flaps. (*) demarcates residual BLSF graft fragments. Scale bars for 1st and 2nd rows in each panel are 3 mm and 600 μm, respectively. **(B)** Top row: Verhoeff Van Gieson (VVG) staining of elastin fibers (arrows) in NSC and regenerated tunica albuginea from BLSF and SIS groups. Bottom row: IHC assessments of collagen type I expression (brown, HRP labeling) in samples described in the top row. Specimens were also counterstained with hematoxylin to visualize nuclei (blue). Scale bars for both rows are 200 µm. **(C)** IHC evaluations of SMA protein expression in specimens detailed in **(A)** with gross (top row) and magnified (boxed) areas of the corpora (bottom row). SMA expression is labeled in red (Alexa Fluor 594 labeling) with DAPI nuclear counterstain displayed in blue. Scale bars for top and bottom rows are 3 mm and 200 μm, respectively. **(D)** Quantitation of total collagen content in corpora of MTS-stained specimens described in **(A)**. **(E)** Quantitation of SMA expression in corpora from groups described in **(C)**. For both panels **(D,E)**, *N* = 3–5 animals per data point were assessed. Values were normalized to NSC levels and are presented as mean ± SD. Data were analyzed with Kruskal Wallis and post-hoc Dunn’s test to determine significance. (Ω) = *p* < 0.05 in comparison to BLSF group. (^§^) = *p* < 0.05 in comparison to NSC. (β) = *p* > 0.05 in comparison to NSC. BLSF, bi-layer silk fibroin scaffold; TV, tunica vaginalis; SIS, small intestinal submucosa; NSC, nonsurgical controls; SMA, α-smooth muscle actin; HRP, horseradish peroxidase.

Histological (MTS, VVG) and IHC (collagen type I, SMA) assessments of NSC and reconstructed penile tissues harvested at 3 months post-op were carried out to characterize the extent of host tissue responses and constructive remodeling at implant regions and corpus cavernosum (Figure 3). The tunica albuginea in NSC was mainly composed of circular layers of collagen type I bundles and fibroblast-like cells interlaced with elastin fibers while the trabeculae of the corpus cavernosum was populated with SMA + smooth muscle cells and blood vessels. Neotissues were observed spanning the original graft site in both SIS and BLSF groups with no substantial contracture noted while the autologous TV flap was mostly intact with minimal ingrowth of surrounding host tissues. The regenerated tunica albuginea in both the SIS and BLSF cohorts contained collagen type I bundles as well as elastin fibers, however collagen organization was qualitatively less developed in reconstructed specimens than observed in NSC. Residual fragments of the BLSF matrix were detected at implant sites surrounded by focal areas of putative macrophage phagocytosis. In contrast, SIS biomaterials were primarily degraded at this endpoint. Scattered mononuclear inflammatory cells were found throughout the graft region of both acellular implants. Histomorphometric analysis of total collagen content in MTS-stained specimens revealed a significant increase in corporal fibrosis in rabbits repaired with SIS grafts (15-fold over NSC; *p* < 0.05) or TV flaps (17-fold over NSC, *p* < 0.05) in respect to NSC. In contrast, total collagen content in the corpora cavernosum of rabbits reconstructed with BLSF matrices was 2.6 fold over NSC levels which was statistically similar between the two groups (*p* > 0.05). In addition, SIS and TV groups respectively displayed 46 and 52% significant reductions (*p* < 0.05) in SMA + corporal smooth muscle cells compared to the BLSF group. These data establish that corporoplasty with BLSF grafts leads to regeneration of tunica albuginea defects with significantly less corporal fibrosis in respect to TV flaps and SIS matrices while maintaining populations of SMA + corporal smooth muscle cells comparable to NSC.

## 4 Discussion

The goal of this study was to assess the feasibility of BLSF matrices for the repair of tunica albuginea defects and compare their performance to conventional TV flaps and SIS biomaterials in a rabbit corporoplasty model. Overall, our results demonstrated that BLSF grafts supported *de novo* formation of tunica albuginea-like tissue composed of collagen type I and elastin fibers at implantation sites. In comparison to TV flaps and SIS matrices, BLSF grafts elicited significantly less corporal fibrosis and preserved levels of smooth muscle cells expressing contractile proteins similar to NSC. Papaverine-induced erectile function was also improved in rabbits repaired with BLSF matrices which displayed a higher proportion of group replicates capable of maintaining full, sustained erections without incidence of perforation in comparison to other study implants. Future preclinical studies will focus on mating evaluations to determine the ability of penile tissues reconstructed with BLSF grafts to penetrate the vaginal vault and facilitate intravaginal ejaculation as well as impregnation. In addition, functional evaluations of BLSF grafts in preclinical models of urologic disease (i.e. Peyronie’s disease) would be desirable prior to clinical translation.

Deficiencies in erectile function in TV and SIS cohorts may be a putative consequence of increased corporal fibrosis and reduction in contractile smooth muscle cells. Indeed, previous studies have significantly correlated these histopathologic factors with erectile dysfunction in patients following radical prostatectomy (Iacono et al., 2005). Moreover, corporal fibrosis has also been linked to elevated levels of oxidative stress and pro-fibrotic cytokines which can stimulate smooth muscle cell apoptosis and excessive collagen deposition resulting in alterations in penile elasticity and compliance (Ferrini et al., 2004). Our findings are consistent with past evaluations of BLSF and SIS matrices in surgical models of esophageal and urethral repair wherein the former elicited less foreign body responses and favored constructive remodeling over scar tissue formation (Chung et al., 2014b; Algarrahi et al., 2015; Algarrahi et al., 2018b). Mechanisms governing penile wound healing patterns following biomaterial implantation are largely unknown. However, differences in regenerative outcomes between BLSF and SIS matrices may be linked to the presence of endogenous transforming growth factor (TGF)-β1 protein found in the latter (Voytik-Harbin et al., 1997). Indeed, TGF-β1 has been previously shown to increase collagen synthesis in corporal smooth muscle cells in culture (Moreland et al., 1995) and be sufficient to induce corporal fibrosis in rabbits *in vivo* (Nehra et al., 1999).

TV flaps were included as a standard of care control group since these tissues are frequently utilized in the pediatric population for correction of hypospadias and penile chordee (Ritchey and Ribbeck, 2003; Braga et al., 2007). Post-operative surveillance of erectile function in boys repaired with TV flaps is limited to short-term studies during preputial and adolescent periods with limited evidence of residual chordee or erectile dysfunction (Ritchey and Ribbeck, 2003; Braga et al., 2007). However, given our current observations that TV flaps elicit elevated levels of corporal fibrosis, prospective studies of long-term outcomes are needed to assess the impact of this surgical approach on erectile function through puberty and into adulthood. Indeed, Harper and colleagues reported that exposure of TV flaps to the external environment and transposition into the urethra can lead to graft retraction and fibrosis (Harper et al., 2017).

## 5 Conclusion

The results from our current study reveal that acellular, BLSF grafts are permissive for regeneration of tunica albuginea defects and can support erectile function in a preclinical animal model. BLSF scaffolds were also found to be superior options for corporoplasty compared to TV flaps and SIS matrices, which elicited significantly higher degrees of corporal fibrosis, incidence of erectile dysfunction, and neotissue perforation during pharmacologically induced erection. In summary, BLSF biomaterials represent attractive candidates for corporoplasty and may offer a functional “off-the-shelf” alternative for the use of conventional autologous tissues for penile repair.

## Data Availability

The original contributions presented in the study are included in the article/supplementary material, further inquiries can be directed to the corresponding authors.
